# Supplementary material for: Chromatin Interaction Changes during the iPSC-NPC Model to Facilitate the Study of Biologically Significant Genes Involved in Differentiation
Source: Genes (Basel). 2020 Oct 8;11(10):1176. doi: 10.3390/genes11101176 (PMC7600115; doi:10.3390/genes11101176)
Supplement: Supplementary file 1 [file genes-11-01176-s001.pdf]

Supplementary Figure 1

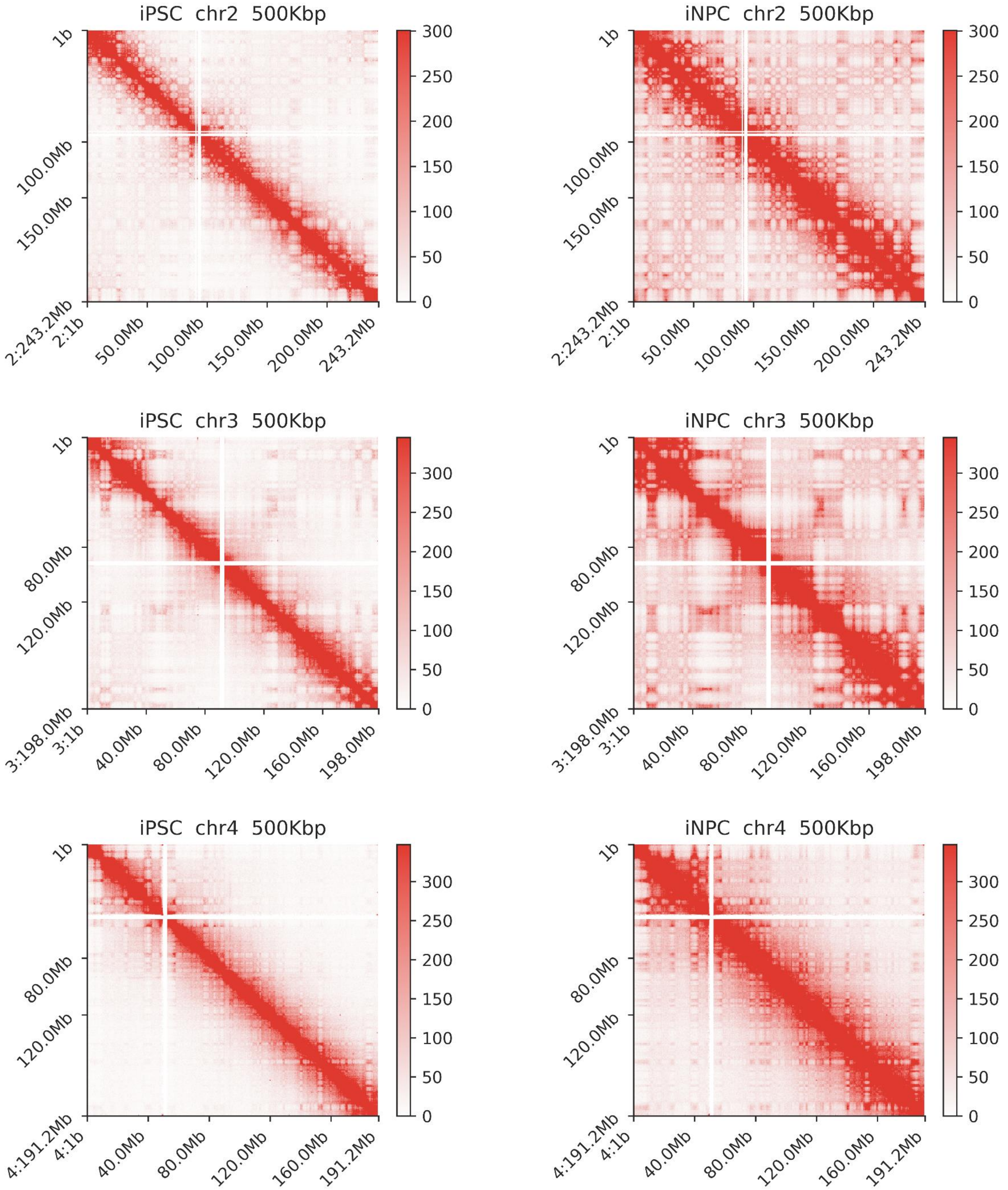

Supplementary Figure 1 (continued)

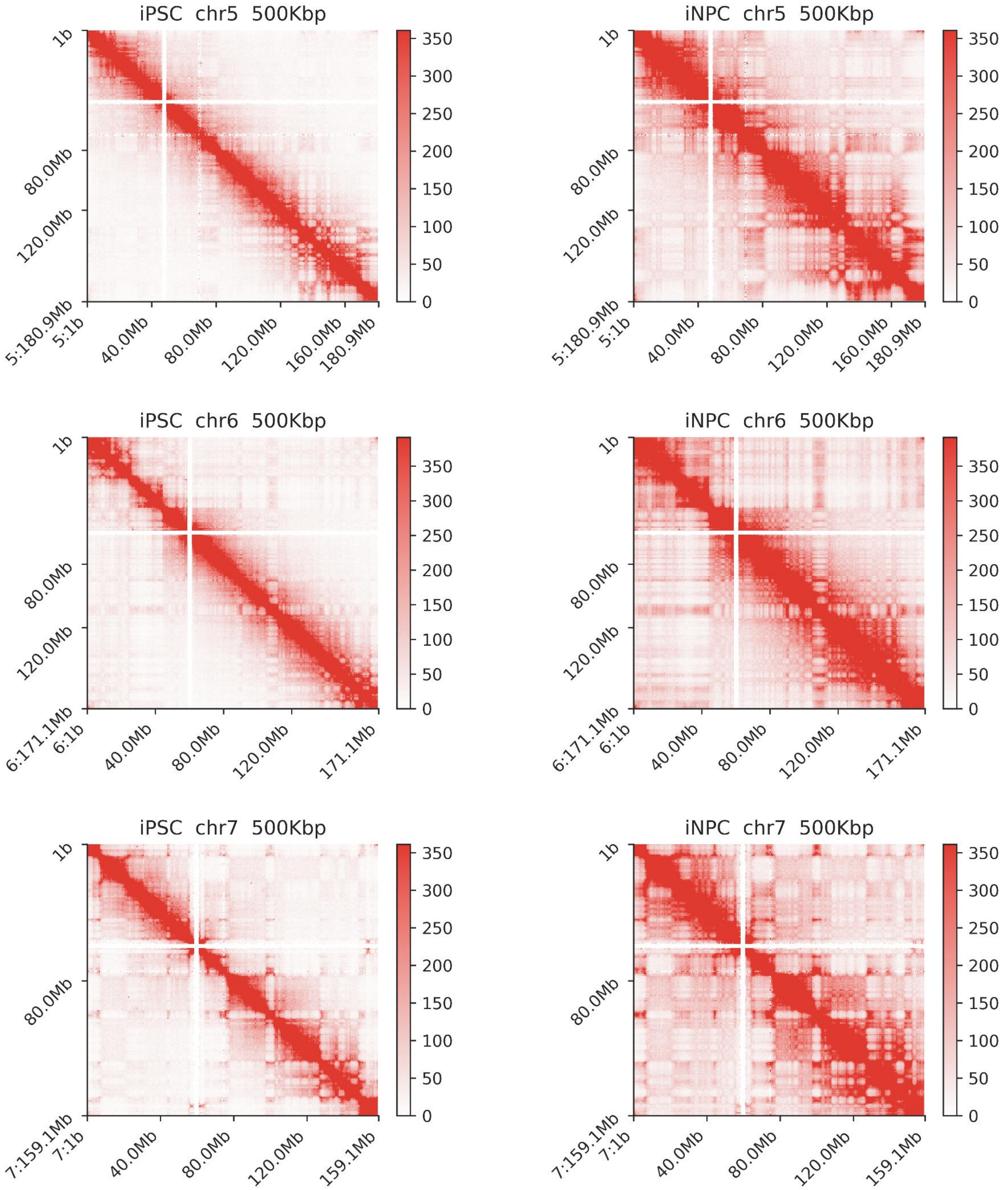

Supplementary Figure 1 (continued)

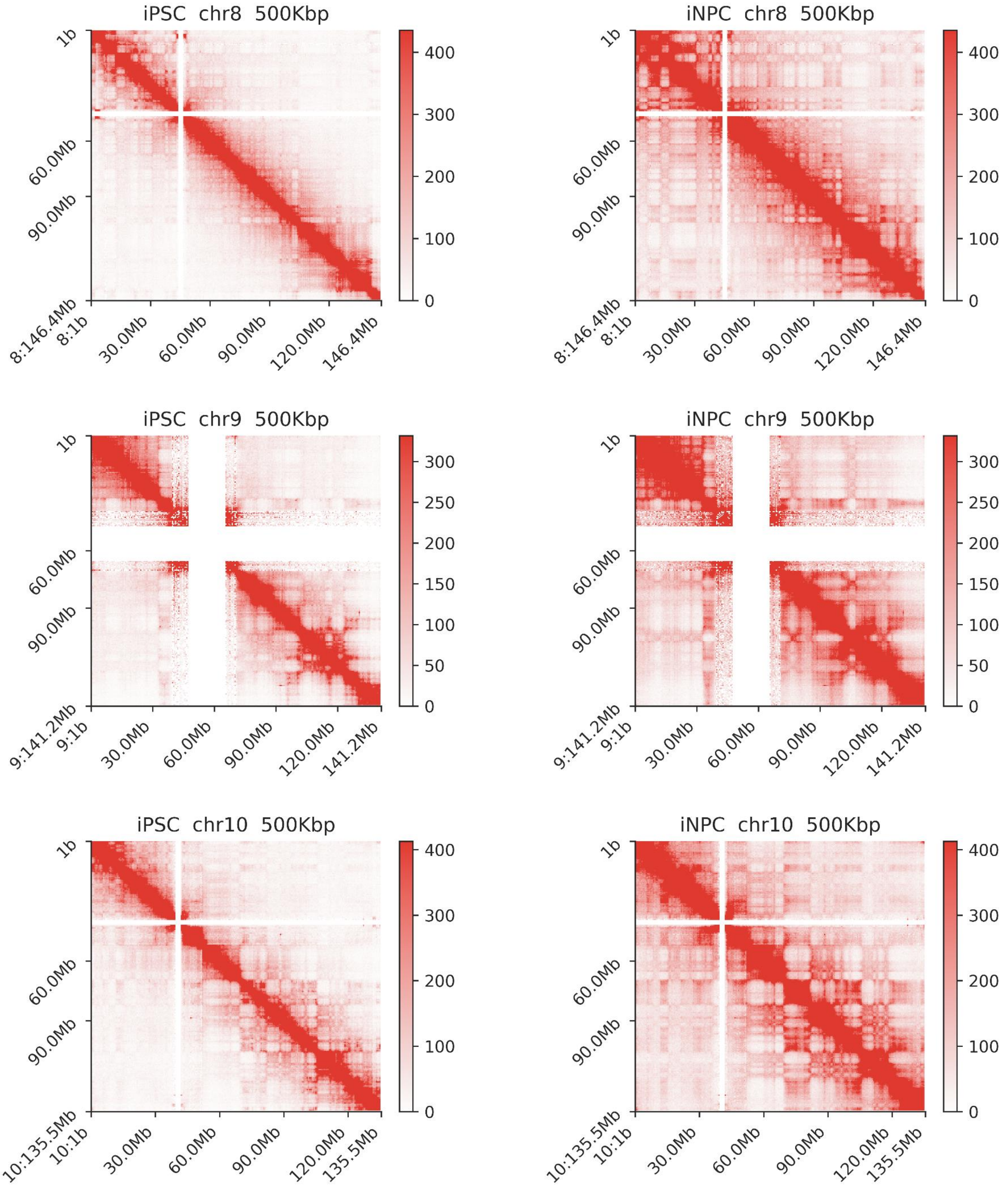

Supplementary Figure 1 (continued)

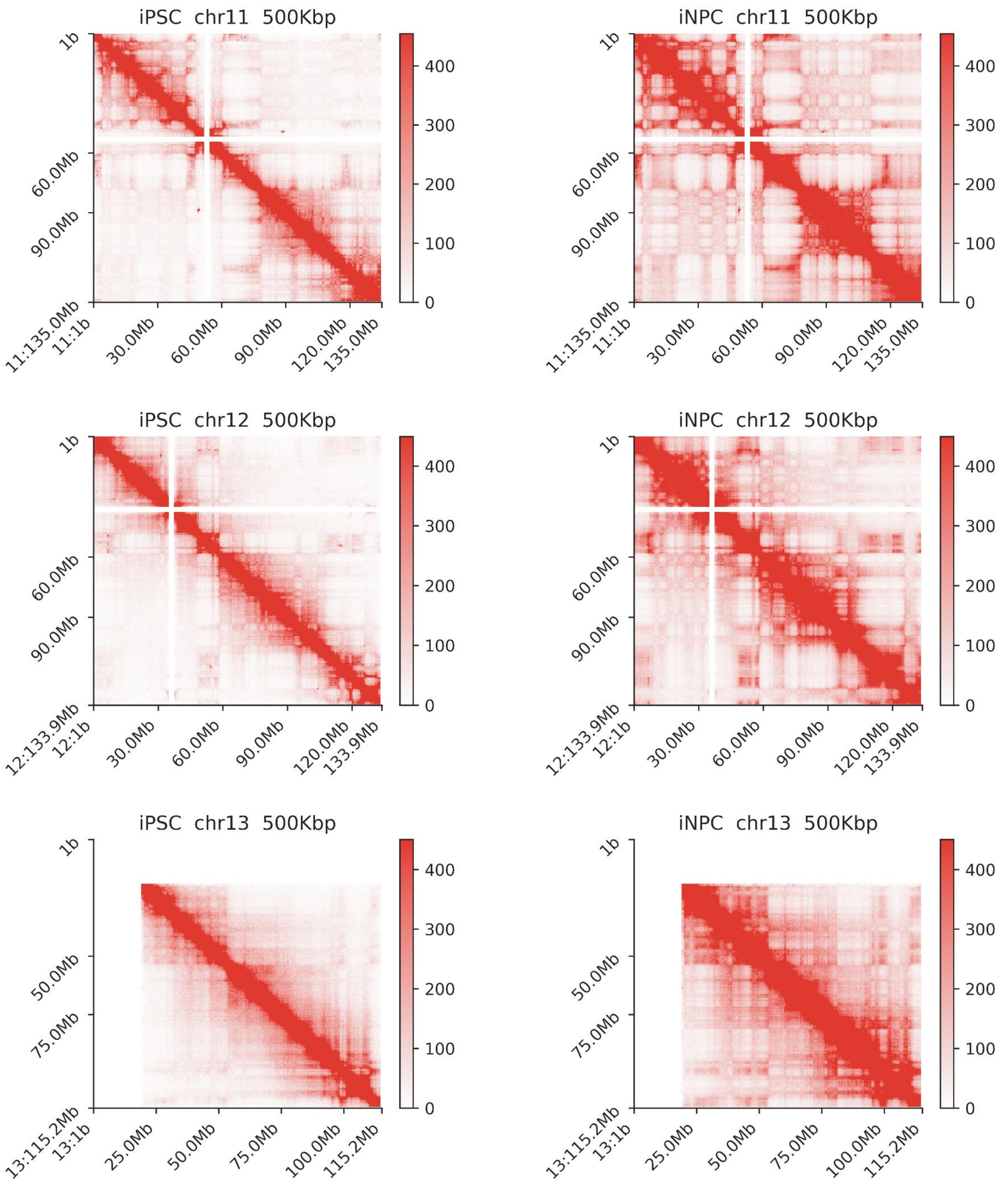

Supplementary Figure 1 (continued)

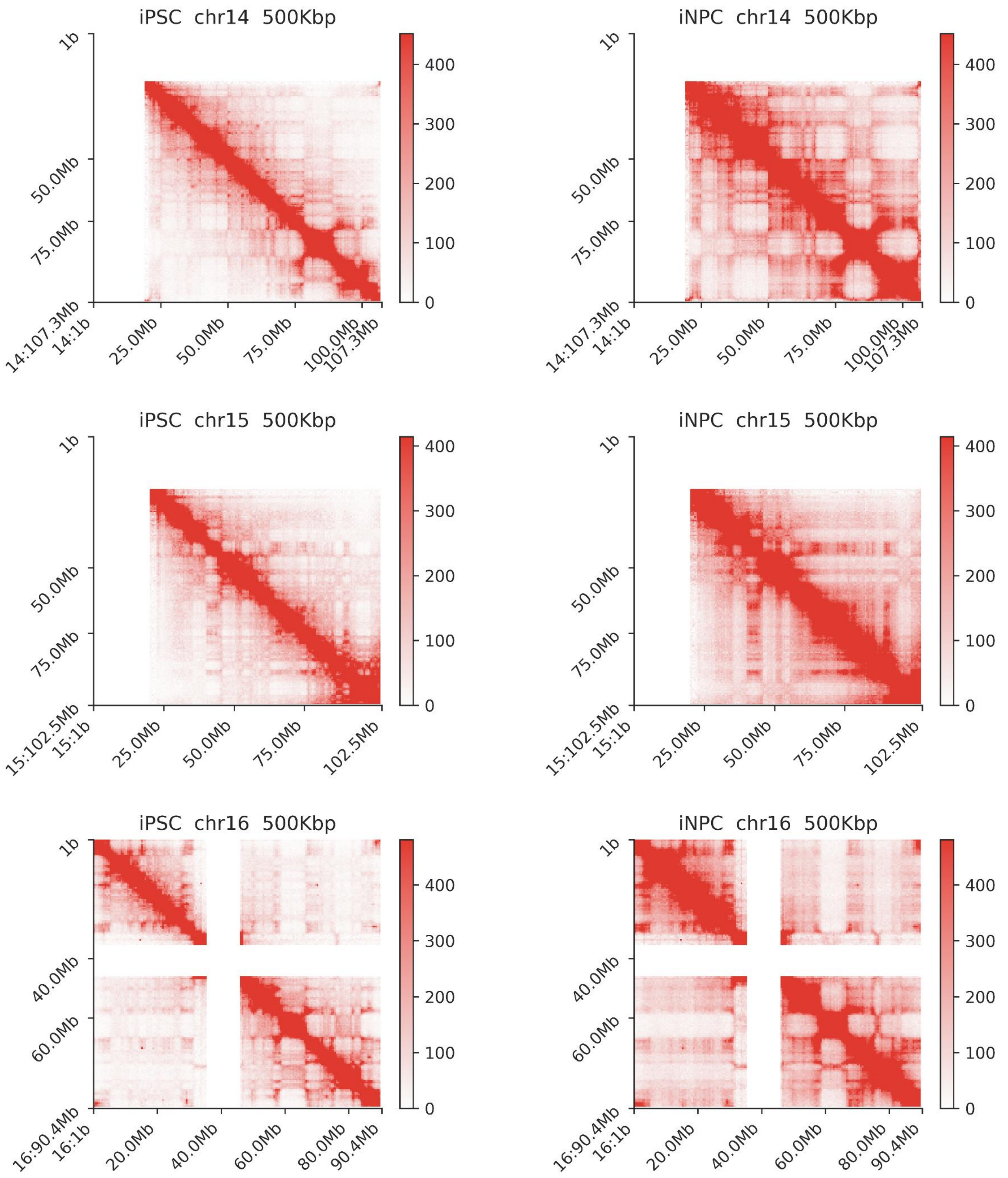

Supplementary Figure 1 (continued)

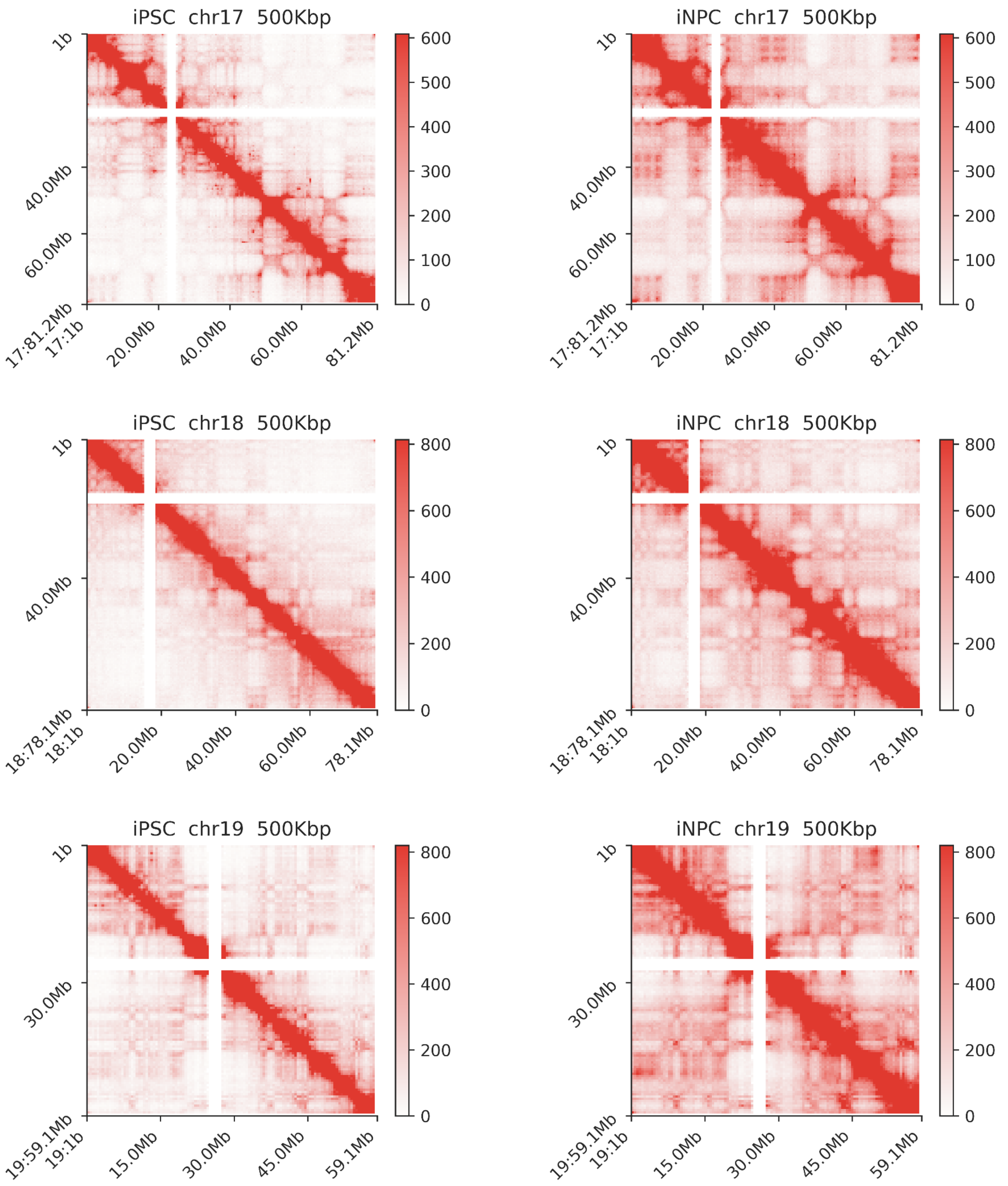

Supplementary Figure 1 (continued)

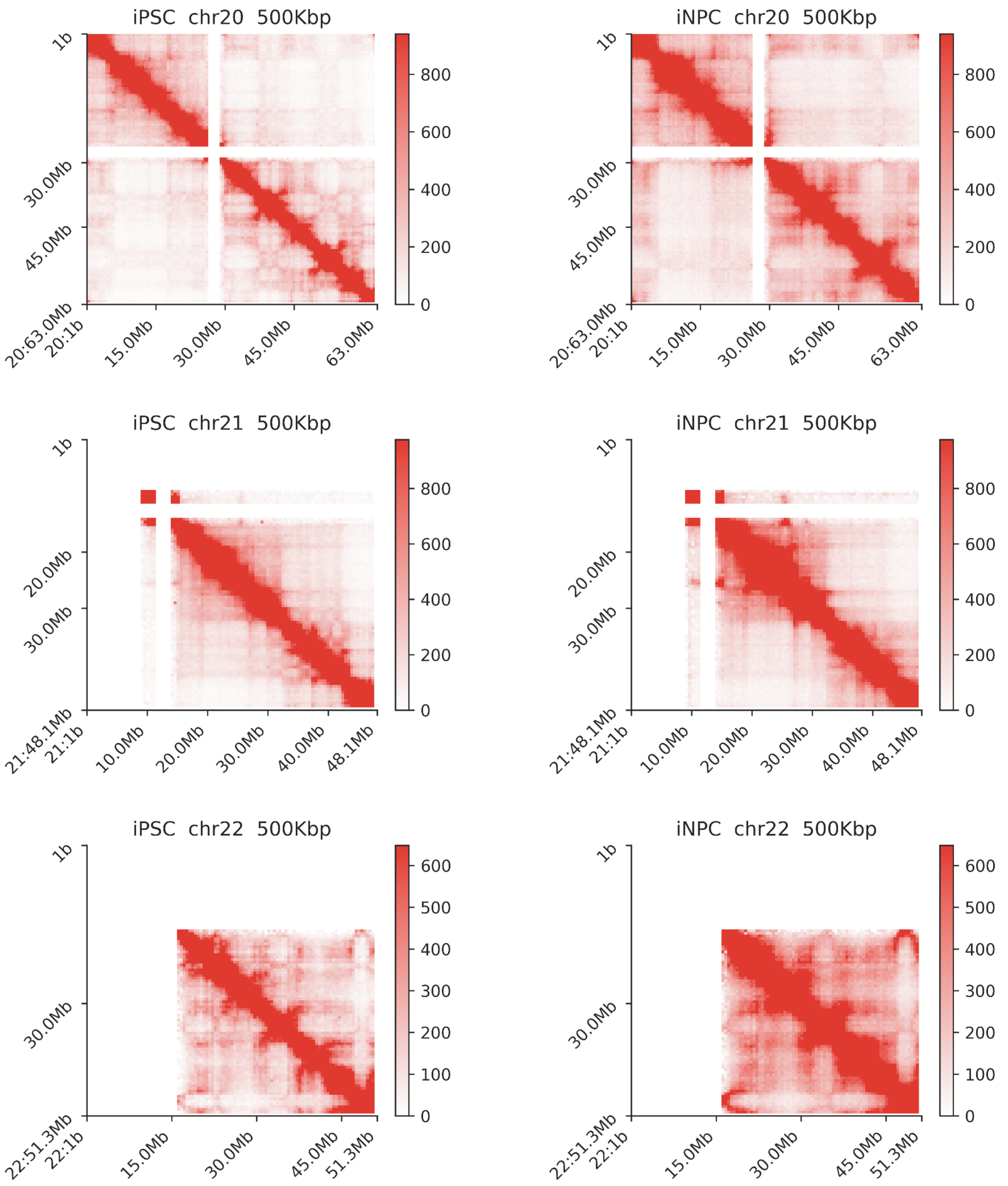

Supplementary Figure 1 (continued)

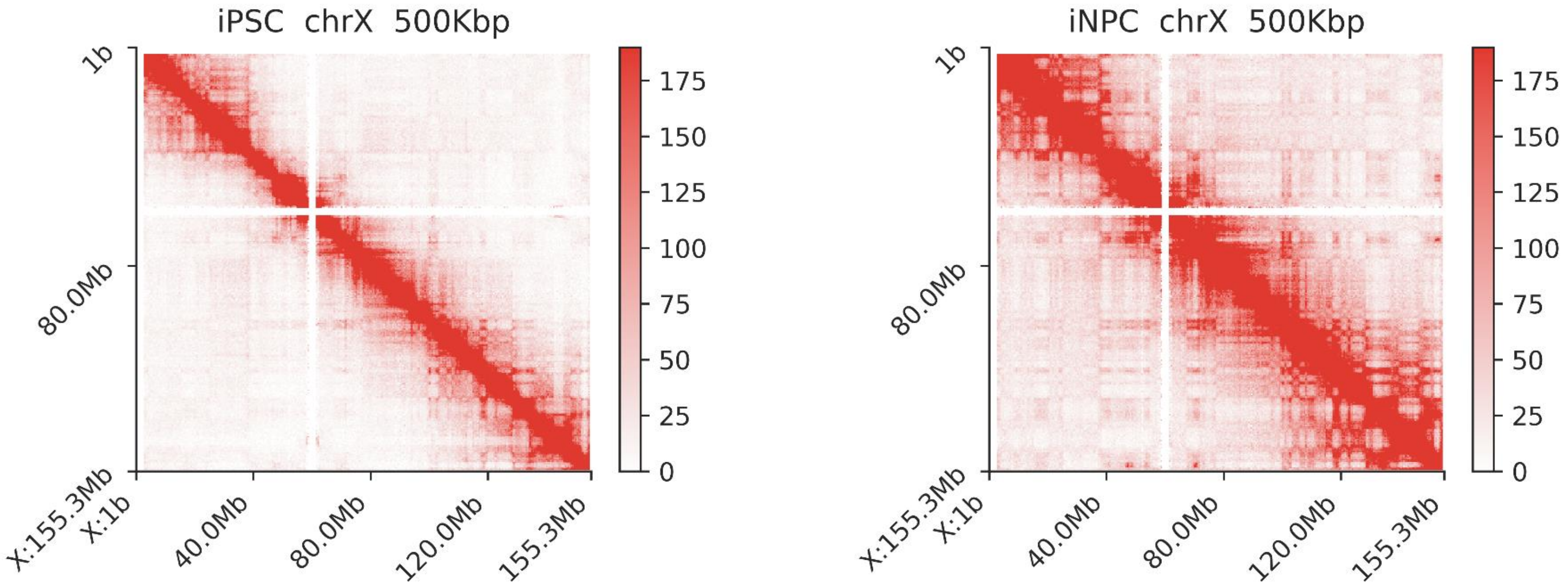

Supplementary Figure 2

A

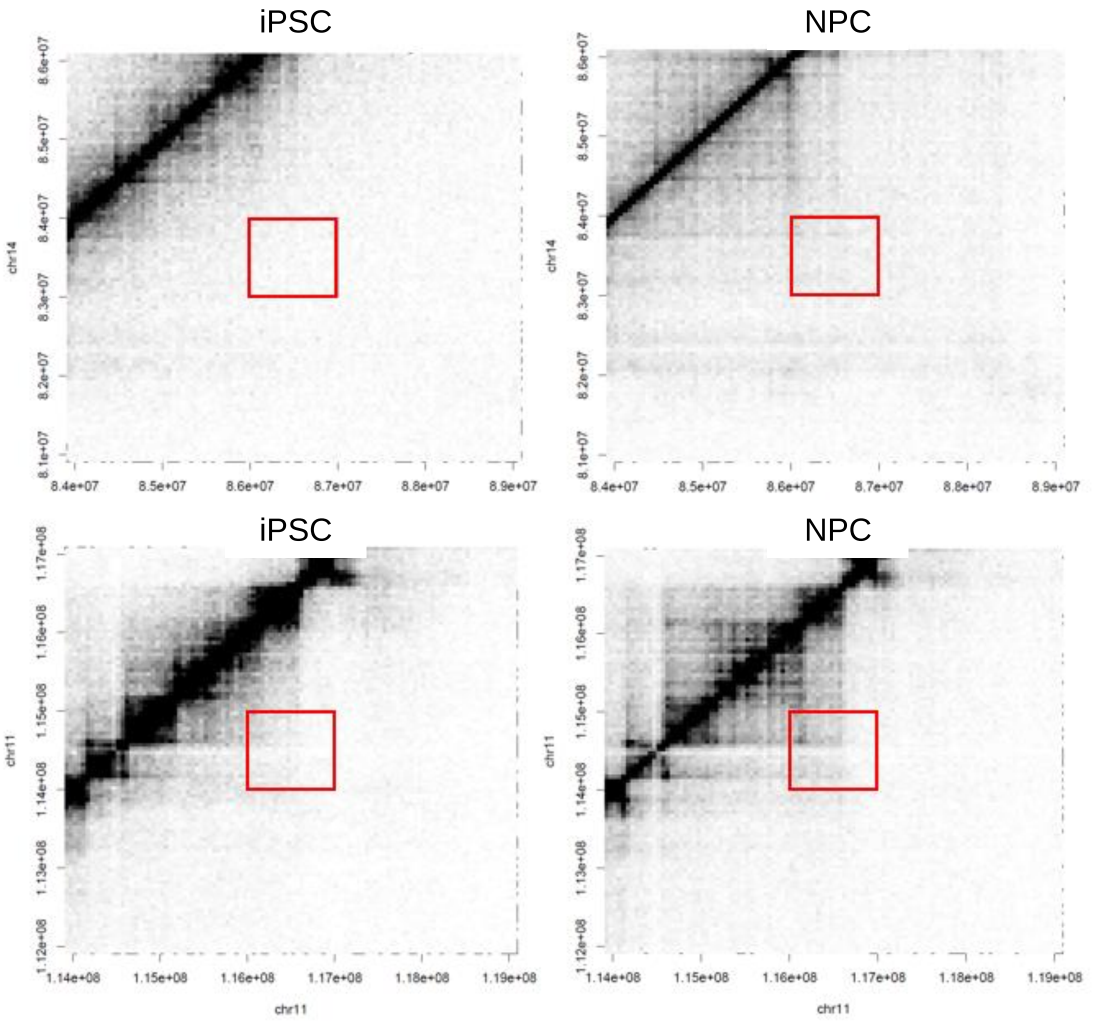

Increased interaction regions during NPC differentiation (FDR<0.01)

B

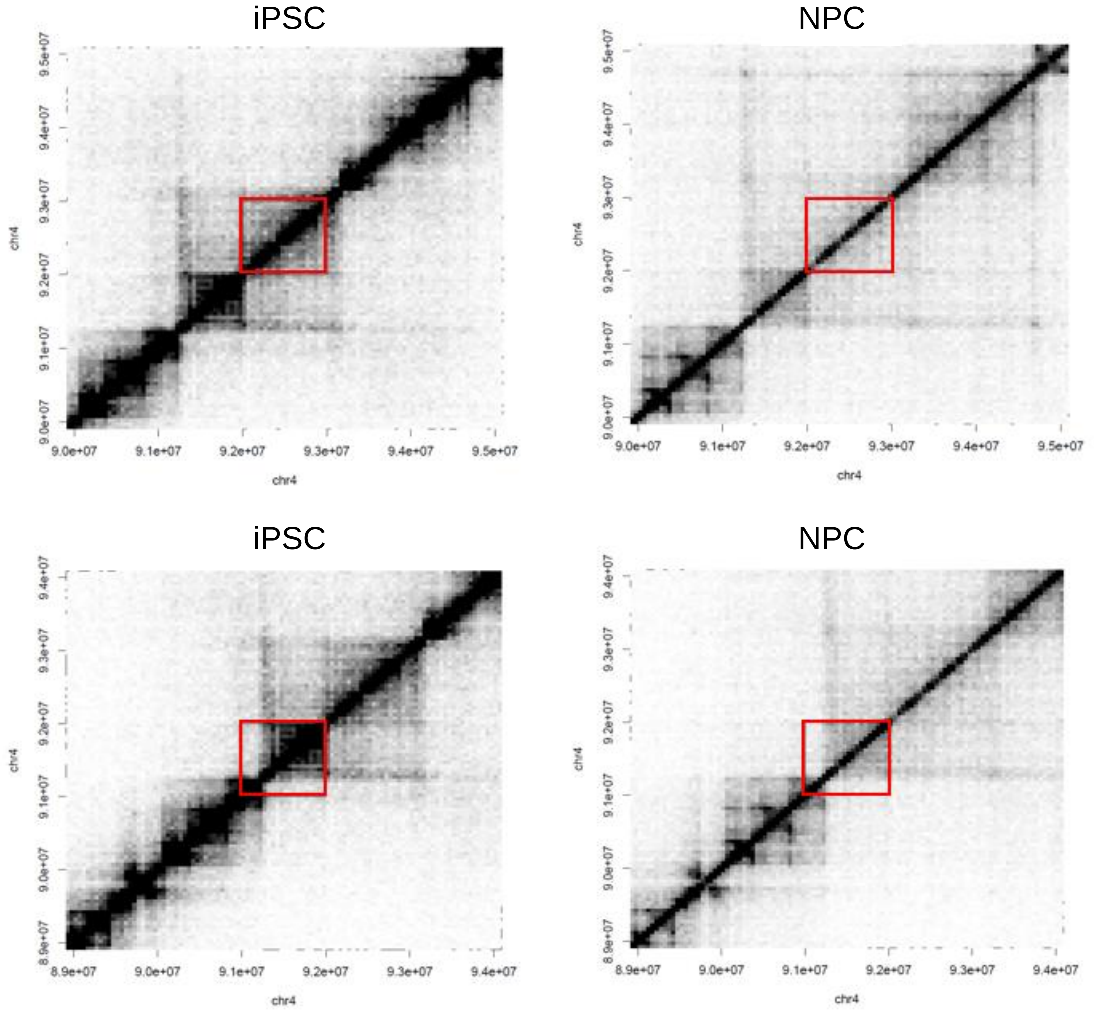

Decreased interaction regions during NPC differentiation (FDR<0.01)

Supplementary Table 1

	Uniquely Mapped Reads
hiPSC	972,325,642
NPC	758,445,782

Supplementary Table 2

Gene ontology analysis of iPSC Group1 and Group2

Promoter-Promoter iPSC Group1	P-Value
G-protein coupled receptor signaling pathway	5.00E-101
detection of chemical stimulus involved in sensory perception	8.80E-20
epidermis development	4.00E-10
keratinocyte differentiation	1.60E-09
cellular defense response	2.00E-08
embryonic skeletal system morphogenesis	4.30E-06
neuron fate specification	1.50E-04
positive regulation of cytokine secretion	2.50E-04
regulation of complement activation	3.30E-04
neuronal action potential	4.00E-04
spinal cord association neuron differentiation	4.30E-04
cell fate determination	9.00E-04
endocrine pancreas development	1.80E-03
proximal/distal pattern formation	2.20E-03
embryonic forelimb morphogenesis	3.00E-03
thyroid gland development	3.70E-03
DNA methylation involved in gamete generation	4.10E-03
pancreas development	5.80E-03

Promoter-Promoter interaction iPSC Group2	P-Value
transcription, DNA-templated	1.00E-13
G2/M transition of mitotic cell cycle	3.90E-07
stem cell population maintenance	1.00E-03
cell cycle arrest	1.80E-03
strand displacement	2.00E-03
mitotic nuclear envelope disassembly	2.60E-03
glycosaminoglycan biosynthetic process	2.60E-03
histone H3 acetylation	4.10E-03
endosome to lysosome transport	4.20E-03
regulation of cyclin-dependent protein serine/threonine kinase activity	4.20E-03
tRNA processing	4.20E-03
negative regulation of MAP kinase activity	6.80E-03

Supplementary Table 3

Gene ontology analysis of NPC Group1 and Group2

Promoter-Promoter interaction NPC Group1	P-Value
detection of chemical stimulus involved in sensory perception of smell	2.00E-109
G-protein coupled receptor signaling pathway	2.50E-107
immune response	3.20E-20
epidermis development	2.90E-11
keratinocyte differentiation	7.00E-09
multicellular organism development	5.10E-08
cellular protein metabolic process	7.00E-08
cell differentiation	2.20E-04
embryonic skeletal system morphogenesis	4.80E-04
anterior/posterior pattern specification	6.30E-04
neuronal action potential	6.70E-04

Promoter-Promoter interaction NPC Group2	P-Value
transcription, DNA-templated	1.10E-23
cell cycle	1.10E-08
neural tube closure	2.10E-06
mRNA splicing, via spliceosome	1.50E-05
transcription initiation from RNA polymerase I promoter	5.10E-05
cell-cell adhesion	1.30E-04
neuron projection development	2.00E-04
positive regulation of protein phosphorylation	2.30E-04
histone deacetylation	2.60E-04
